# Familism and its Impact on Younger African-American Informal Family Caregiver Role Strain and Decision-Making

**DOI:** 10.1093/geroni/igaa057.2253

**Published:** 2020-12-16

**Authors:** Ivana Alexander

**Affiliations:** University of Maryland Baltimore, Baltimore, Maryland, United States

## Abstract

Informal family caregivers of older adults are the life’s blood of the long-term services and supports (LTSS) system in the United States, providing an estimated $470 billion in unpaid care each year. This care is disproportionately provided by racial and ethnic minority families, where systemic economic disparities make it impossible to afford formal care in many cases. Adding to this are the cultural expectations or familism values that often influence attitudes and beliefs about caregiving. These expectations and values contribute to the emotional, psychological, financial, and professional strain experienced by caregivers. They also impact the caregiver-healthcare provider dyad as well as the caregiver-patient-healthcare provider triad, where shared decision-making is an essential component of providing LTSS for family members. This presentation applies an auto-enthographic lens to the author’s personal experiences as a younger African-American female caregiver of an elderly, terminally ill family member and as a medical social worker to highlight the gaps that exist between familism values, the shifting reality of family caregiving, and available resources and addresses related implications for the future of long-term care in a family-centered context.

